# Descriptions of two new species of the genus *Camaena* from Guangxi, China (Gastropoda, Stylommatophora, Camaenidae)

**DOI:** 10.3897/zookeys.634.10236

**Published:** 2016-11-21

**Authors:** Hong-Mu Ai, Jun-Hong Lin, Pei Wang, Wei-Chuan Zhou, Chung-Chi Hwang

**Affiliations:** 1 College of Plant Protection, Fujian Agriculture and Forestry University, Fuzhou, Fujian 350002, China; 2 Key Laboratory of Molluscan Quarantine and Identification of AQSIQ, Fujian Entry-Exit Inspection & Quarantine Bureau, Fuzhou, Fujian 350001, China; 3 Department of Life Sciences, National University of Kaohsiung, No.700, Kaohsiung University Road, Nan-Tzu District, Kaohsiung 81148, Taiwan

**Keywords:** Camaena detianensis sp. n., Camaena lingyunensis sp. n., camaenid species, molecular phylogeny, taxonomy

## Abstract

The sinistral *Camaena* species are mainly distributed in southern China and northern Vietnam. There is a total of eight species or subspecies of sinistral *Camaena* recorded at present. By systematically collecting specimens in Guangdong, Guangxi and Yunnan in southern China and the northern areas in Vietnam, two new species, *Camaena lingyunensis* Zhou & Lin, **sp. n.** and *Camaena detianensis* Zhou & Lin, **sp. n.** have been discovered. These new species are here characterised based on the comparison of shells, their reproductive system, the molecular phylogenetic analyses of the mitochondrial genes COI and 16S, and the nuclear gene ITS2. Detailed descriptions of the morphological characters, the DNA sequences, and the habitat of the two new species are given. Differential comparisons with related species are provided as well as a key to the sinistral species of *Camaena*.

## Introduction

The genus *Camaena*, which contains large dextral or sinistral shell, was established in 1850 by Albers, with the type species *Helix cicatricosa* Müller, 1774. The common features of shell include a large protoconch, a scar-like protuberance or malleation on surface, tawny colouring, and multiple red or brown spiral bands.

The classification of this genus is confused historically, which is especially true for *Camaena cicatricosa* (Müller, 1774) in the sinistral group. Most taxonomists divided the sinistral group, which is distributed in southern China and northern Vietnam into three species, *Camaena cicatricosa*, *Camaena hahni* (Mabille, 1887), and *Camaena seraphinica* (Heude, 1890). The taxonomic statuses of *Camaena hahni* and *Camaena seraphinica* are relatively stable. *Camaena hahni* contained two subspecies, *Camaena hahni hahni* and *Camaena hahni broti* (Dautzenberg & d’Hamonville, 1887). Some scholars considered as a synonym of *Camaena hahni* (e.g. Pilsbry 1891). The classification of *Camaena cicatricosa* is most confused, and different western scholars divided it into five distinct subspecies or variations on the basis of diverse shell, *Camaena cicatricosa cicatricosa*, *Camaena cicatricosa ducalis* (Ancey, 1885), *Camaena cicatricosa inflata* (Möllendorff, 1885), *Camaena cicatricosa obtecta* (Fischer, 1898), and *Camaena cicatricosa connectens* (Dautzenberg & Fischer, 1906). However, due to lack of fresh specimens, further research on the histological anatomy and molecular biology have not been done. The taxonomic status of the sinistral group has always been controversial, and scientific names have been revised repeatedly. Previously, the Chinese scholars disagreed with this classification, and the name *Camaena cicatricosa* was used (e.g. Chen and Gao 1987). Ding et al. (2016) clarified the phylogenetic relationships and taxonomic status of the sinistral group with the help of comparative shell morphology, genital anatomy, and molecular phylogeny, and recognised it to contain four species, *Camaena cicatricosa*, *Camaena inflata* (Möllendorff, 1885), *Camaena obtecta* (Fischer, 1898) and *Camaena connectens* (Dautzenberg & Fischer, 1906). In addition, they described one new species *Camaena poyuensis* (Zhou, Wang & Ding, 2016). Thus, the number of species within the sinistral *Camaena* group now reached eight species or subspecies.

On the basis of the above work, the authors have studied a large number of specimens collected in Guangdong, Guangxi and Yunnan in southern China and the northern areas in Vietnam during 2013–2016, and discovered two new species according to shell morphology, reproductive system, and molecular biology. The details including morphological characteristics, DNA sequences, and habitat of these two new species are described herein.

## Material and methods

**Figure 1.**
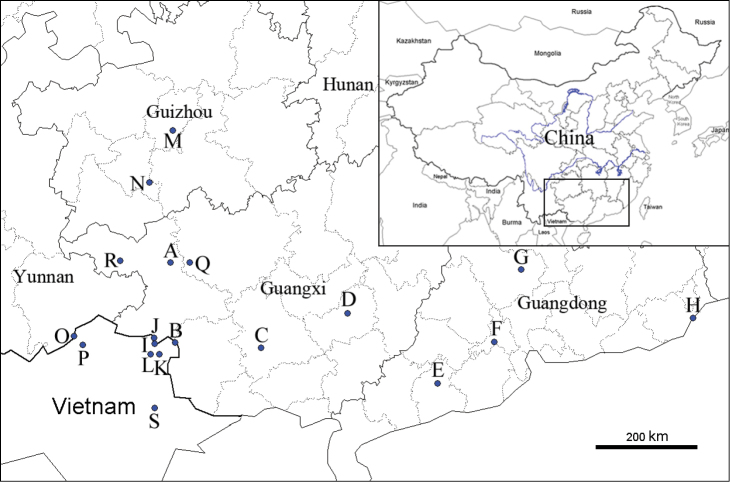
Map of locations of *Camaena* species. *Camaena lingyunensis* sp. n.: **A** Kasuo, Lingyun, Guangxi, China. *Camaena detianensis* sp. n.: **B** Detian Falls, Daxin, Guangxi, China. *Camaena cicatricosa*: **C** Nanning, Guangxi, China **D** Guiping, Guangxi, China **E** Yangchun, Guangdong, China **F** Gaoming, Canton, Guangdong, China **G** Yingde, Guangdong, China **H** Shantou, Guangdong, China. *Camaena obtecta*: **I** Buhaitun, Jinxi, Guangxi, China **J** Longbang, Jingxi, Guangxi, China **K** Longo coc tan, Quang-Huyen, Vietnam **L** Cao Bang, Vietnam (type locality). *Camaena inflata*: **M** Qianlin park, Guiyang, Guizhou, China **N** Ziyun, Anshun, Guizhou, China. *Camaena connectens*: **O** Tianbao, Malipo, Yunnan, China **P** Ha Giang, Vietnam (type locality). *Camaena poyuensis*: **Q** Poyue, Bama, Hechi, Guangxi, China. *Camaena seraphinica*: **R** Dingan, Tianlin, Guangxi, China (type locality). *Camaena hahni*: **S** Huu Lien Nature Reserve, Lang-Son, Vietnam.

This study is based on material collected by the authors from several sites in China (Fig. 1). The longitude and latitude were recorded using a GPS. The live adults were drowned in water for 12–24 hours, and then killed in hot water. Soft body parts were preserved in 75% or 95% ethanol and stored at -20°C. Empty shells were cleaned and preserved at room temperature. Samples have been deposited in the State Key Laboratory of Molluscan Quarantine and Identification, FJIQBC.

Shells were measured to 0.1 mm using electronic calipers. Standard shell parameters were taken following Kerney and Cameron (1979). All adult specimens of each species were measured. Only sexually matured specimens were dissected for the examination of reproductive system. Genitalia were dissected under a dissecting microscope (ZEISS Stemi 2000); three specimens of each species were dissected. Terminology for reproductive system follows Gómez (2001). All drawings were traced with the aid of a Canon 550D digital camera.

Approximately 0.02–0.04 g of foot muscle tissue was used for DNA extraction. The muscle tissue was bathed in sterile water for 3–6 hours to remove residual alcohol. Genomic DNA was isolated using Qiagen DNeasy Blood & Tissue kit (Qiagen, Beijing) on more than three specimens per species, examined by agarose gel electrophoresis, and stored at -20°C for further use. The partial mitochondrial cytochrome c oxidase subunit 1 (COI) and 16S rRNA (16S), and the internal transcribed spacer 2 (ITS2) region of nuclear ribosomal DNA were amplified by PCR using the primer pairs, reaction systems and amplification conditions listed in Table 1. The PCR products were analysed by 1.2% agarose gel electrophoresis.

**Table 1.** Primer pairs and PCR conditions used in the analyses of the COI, 16S rRNA, and ITS2 genes of *Camaena*.

Gene	COI
Primer pairs (5’-3’)	LCO:GGTCAACAAATCATAAAGATATTGG
HCO:TAAACTTCAGGGTGACCAAAAAATCA	
Reaction systems	25ul Taq PCR MasterMix×2; 1ul each primer; 2ul DNA; 16ul ddH_2_O
Cycling conditions	94°C: 30s; 94°C: 10s, 45°C: 50s, 72°C: 1min, 40 cycles; 72°C: 10min.
Reference	Folmer et al. 1994
Gene	16S
Primer pairs (5’-3’)	16SAR:CGCCTGTTTATCAAAAACAT
16SBR:CCGGTCTGAACTCAGATCACGT	
Reaction systems	25ul Taq PCR MasterMix×2; 1ul each primer; 2ul DNA; 16ul ddH_2_O
Cycling conditions	94°C: 30s; 94°C: 10s, 45°C: 50s, 72°C: 1min50s, 40 cycles; 72°C: 10min.
Reference	Palumbi et al. 1991
Gene	ITS2
Primer pairs (5’-3’)	FYIT2:CATCGACATCTTGAACGCACAT
RYIT2:TCCCAAACAACCCGACTCCT	
Reaction systems	25ul Taq PCR MasterMix×2; 1ul each primer; 2ul DNA; 16ul ddH_2_O
Cycling conditions	94°C: 30s; 94°C: 10s, 55°C: 30s, 72°C: 1min30s, 40 cycles; 72°C: 10min.
Reference	Ding et al. 2016

After sequencing, raw sequences were proof-read on chromatograms and aligned into contigs using BioEdit 7.2 (Hall 1999). ITS2 sequences were annotated by using HMMer (Eddy 1998) and ITS2 Database (Koetschan et al. 2010). Sequence alignments were generated using ClustalW implemented in MEGA 5 (Tamura et al. 2011). A total of 165 sequences of COI, 16S, and ITS2 were used in this study, 36 sequences of which were newly generated and deposited in GenBank (Table 2), and the rest referenced in Ding et al. (2016). Pairwise *p*-distances between taxa were calculated using MEGA5. For phylogenetic analysis, the three sequenced data sets were concatenated into one, with a length of 1,619 bp. The concatenated alignment contained 39 unique sequences, which were used for subsequent analysis. Neighbor Joining (NJ), Maximum Parsimony (MP), and Maximum Likelihood (ML) analyses based on COI+16S+ITS2 combined data set were performed using MEGA5 with default settings. *Bradybaena sequiniana* (Heude,1885) and *Cornu aspersum* (Müller, 1774) were used as outgroups. The node support values were assessed by bootstrap resampling (Felsenstein 1985) using 1000 replicates.

**Table 2.** Sampling information and GenBank accession numbers of some species.

Sampling	Locality	Collection date	Coordinates	Accession number		
				COI	16S rRNA	ITS2
*Camaena lingyunensis* sp. n.	Kasuo, Lingyun, Guangxi, China	2014.04.24	24°17'47.33"N, 106°39'6.53"E	KX345077 KX345078 KX345079	KX345083 KX345084 KX345085	KX345089 KX345090 KX345091
*Camaena detianensis* sp. n.	Detian Falls, Daxin, Guangxi, China	2013.05.21	22°51'29.54"N, 106°43'13.51"E	KX345074 KX345075 KX345076	KX345080 KX345081 KX345082	KX345086 KX345087 KX345088
*Camaena hahni*	Huu Lien Nature Reserve, Lang-Son, Vietnam	2016.06.22	21°44'53.28"N, 106°22'57.96"E	KX621263 KX621264 KX621265	KX621257 KX621258 KX621259	KX621269 KX621270 KX621271
*Camaena obtecta*	Longo coc tan, Quang-Huyen, Vietnam	2016.06.20	22°41'19.86"N, 106°26'16.50"E	KX621260 KX621261 KX621262	KX621254 KX621255 KX621256	KX621266 KX621267 KX621268

### Abbreviations used

COI
 cytochrome c oxidase subunit 1 gene 


16S
16S rRNA gene 


ITS2
 internal transcribed spacer 2 region of nuclear ribosomal DNA 


NJ
 Neighbor Joining 


ML
 Maximum Likelihood 


FJIQBC
 Fujian Entry-Exit Inspection & Quarantine Bureau, Fuzhou, Fujian, China 


MNHN
 Muséum National d’Histoire Naturelle, Paris, France 


MP
 Maximum Parsimony 


## Results

### Molecular analysis

Molecular analysis was based on DNA sequences of 53 specimens in the genus *Camaena* from 18 localities. In this study a total of 165 sequences of COI, 16S and ITS2 was used. There were 36 sequences from *Camaena lingyunensis* sp. n., *Camaena detianensis* sp. n., *Camaena obtecta* (Fischer, 1898) (distributed in Longo coc tan, Quang-Huyen, Cao Bang, Vietnam) and *Camaena hahni* (distributed in Huu Lien Nature Reserve, Huu Lung, Lang-Son, Vietnam) listed in Table 2. The rest of the sequences and geographical information from five sinistral *Camaena* (*Camaena cicatricosa*, *Camaena obtecta*, *Camaena inflata*, *Camaena connectens* and *Camaena poyuensis*), two dextral *Camaena* (*Camaena menglunensis* and *Camaena jingpingensis*) and the outgroup (*Bradybaena sequiniana* and *Cornu aspersum*) were taken from a previous article (Ding et al. 2016). The sequence alignment was based on lengths of 601 bp (COI), 428 bp (16S) and 590 bp (ITS2), respectively.

Inter- and intraspecific *P*-distances from the three genes of eight species were calculated and listed in Table 3. According to the results of target gene COI, the *p*-distances between *Camaena lingyunensis* sp. n. and other seven sinistral *Camaena* were 0.098–0.178, and the *p*-distances between *Camaena detianensis* sp. n. and other seven sinistral *Camaena* were 0.073–0.189. These numbers significantly exceed the interspecific differentiation standard of terrestrial molluscs, the limit of *p*-distance 0.03 (average 0.03, generally between 0.00–0.06) (Criscione and Köhler 2014).

**Table 3.** Inter and intraspecific *P*-distances of sinistral *Camaena* species.

*p*-distance								
	*Camaena lingyunensis* sp. n.		*Camaena detianensis* sp. n.		*Camaena hahni*		*Camaena cicatricosa*	
	within	between	within	between	within	between	within	between
COI	0.000 -0.002	0.098 -0.178	0.000 -0.002	0.073 -0.189	0.000 -0.004	0.119 -0.183	0.000 -0.017	0.073 -0.168
16S	0.000 -0.005	0.046 -0.145	0.000 -0.002	0.013 -0.137	0.000 -0.002	0.094 -0.153	0.000 -0.016	0.013 -0.137
ITS2	0.000	0.008 -0.045	0.000	0.000 -0.044	0.002 -0.004	0.025 -0.068	0.000 -0.006	0.000 -0.048
	*Camaena poyuensis*		*Camaena connectens*		*Camaena inflata*		*Camaena obtecta*	
	within	between	within	between	within	between	within	between
COI	0.000 -0.003	0.104 -0.177	0.000	0.108 -0.179	0.000 -0.019	0.155 -0.185	0.000 -0.010	0.154 -0.189
16S	0.002 -0.007	0.062 -0.160	0.000 -0.002	0.086 -0.157	0.000 -0.007	0.093 -0.141	0.000 -0.007	0.122 -0.160
ITS2	0.000	0.025 -0.057	0.006 -0.015	0.008 -0.068	0.000	0.008 -0.042	0.000 -0.002	0.008 -0.055

**Figure 2.**
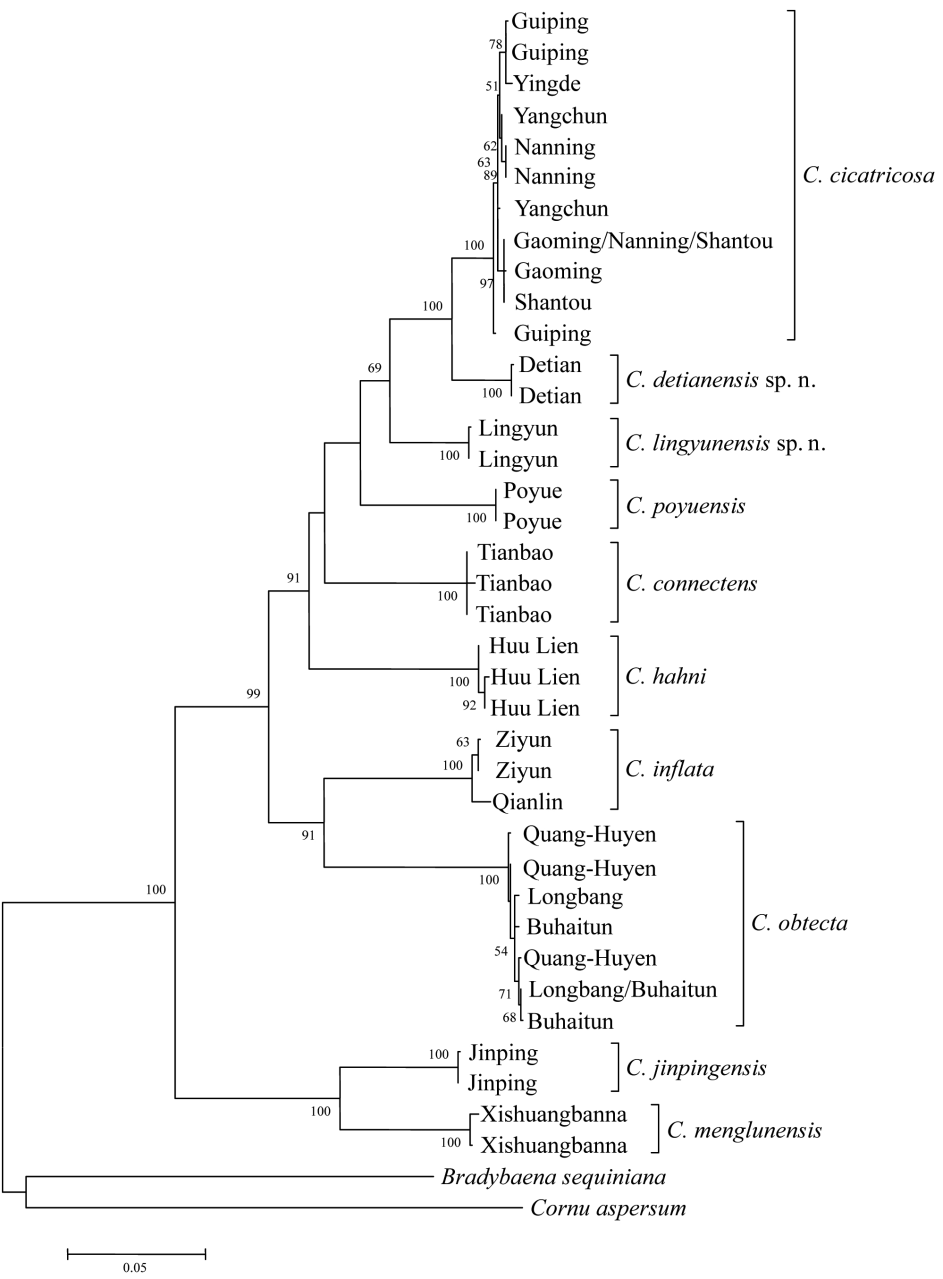
Maximum Likelihood tree based on analysis of the concatenated dataset of COI, 16S, and ITS2 sequences. Numbers beside nodes indicate bootstrapping support (%) for main clades.

The phylogenetic analysis showed that NJ, MP, and ML trees have the mostly same topological structure, and indicated that phylogenetic analyses in this research was relatively correct and reliable, and can be applied in genetic relationship research and systematic classification. The support degree of each species on ML tree (Fig. 2) all reached 100, and eight clades contained described and published species (including 2 dextral species as contrast), and another two clades included two new taxon. In this study, the genus *Camaena* was clearly divided into sinistral and dextral groups. From the tree structure, branch length and comparison of the known species, the phylogenetic tree supported *Camaena lingyunensis* sp. n. and *Camaena detianensis* sp. n. as new species. Moreover, the two new species have closer genetic relationship with *Camaena cicatricosa*, all of three have semi-open or open umbilicus.

## Systematics

### Camaenidae Pilsbry, 1895

#### Camaena

Taxon classificationAnimaliaStylommatophoraCamaenidae

Albers, 1850

##### Type species.

*Helix cicatricosa* Müller, 1774, subsequent designation by Martens, 1860.

#### Camaena lingyunensis

Taxon classificationAnimaliaStylommatophoraCamaenidae

Zhou & Lin sp. n.

http://zoobank.org/9A26F678-0F74-42FC-A37A-610668FDEBB7

**Figure 3.**
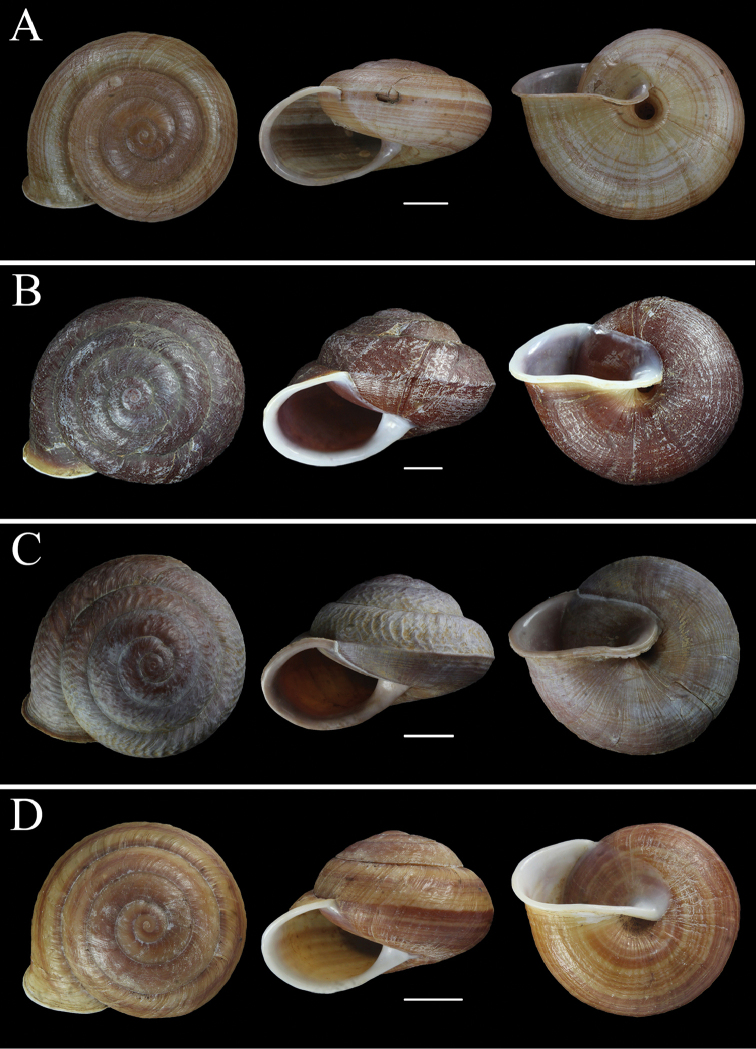
Photographs of shells. **A** *Camaena lingyunensis* sp. n. (holotype, FJIQBC 19280, Kasuo, Lingyun, Guangxi, China) **B** *Camaena detianensis* sp. n. (holotype, FJIQBC 18472, Detian Falls, Daxin, Guangxi, China) **C** *Camaena hahni* (FJIQBC 19300, Huu Lien Nature Reserve, Lang-Son, Vietnam) **D** *Camaena cicatricosa* (FJIQBC 18505, Guiping, Guangxi, China). Scale bars 10 mm.

**Figure 4.**
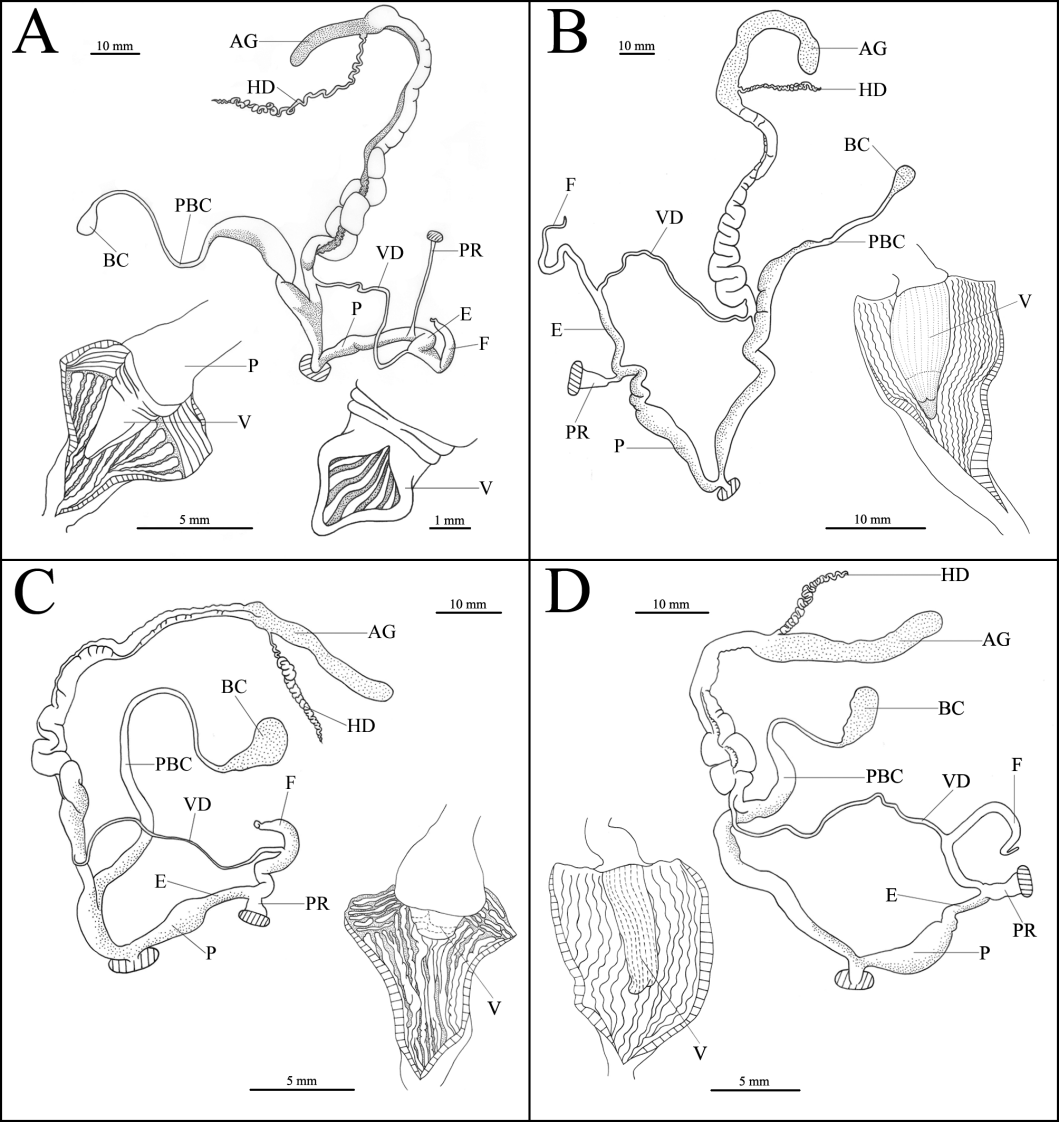
Reproductive system. **A** *Camaena lingyunensis* sp. n. (holotype, FJIQBC 19280, Kasuo, Lingyun, Guangxi, China) **B** *Camaena detianensis* sp. n. (holotype, FJIQBC 18472, Detian Falls, Daxin, Guangxi, China) **C** *Camaena hahni* (FJIQBC 19301, Huu Lien Nature Reserve, Lang-Son, Vietnam) **D** *Camaena cicatricosa* (FJIQBC 18505, Guiping, Guangxi, China). Abbreviations: V, verge; AG, albumen gland; BC, bursa copulatrix; E, epiphallus; F, flagellum; HD, hermaphroditic duct; P, penis; PR, penis retractor muscle; PBC, pedunculus of bursa copulatrix; VD, vas deferens.

**Figure 5.**
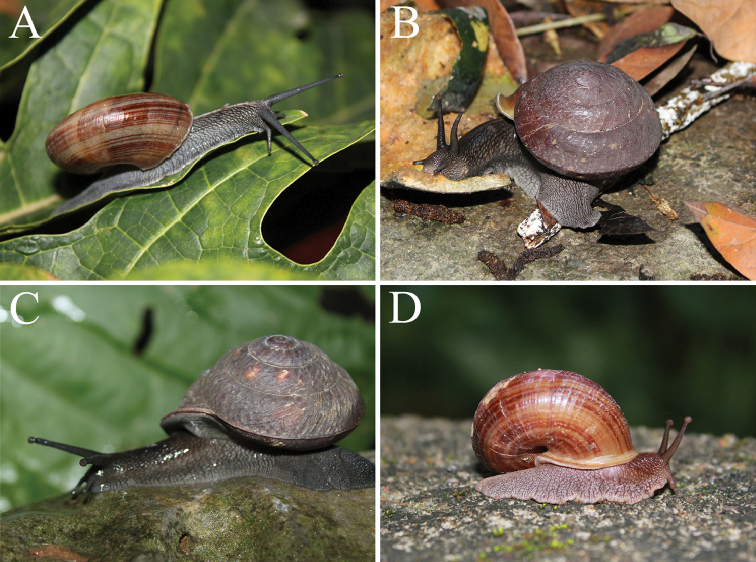
Ecological photographs of snails. **A** *Camaena lingyunensis* sp. n. (Kasuo, Lingyun, Guangxi, China) **B** *Camaena detianensis* sp. n. (Detian Falls, Daxin, Guangxi, China) **C** *Camaena hahni* (Huu Lien Nature Reserve, Lang-Son, Vietnam) **D** *Camaena cicatricosa* (Yangchun, Guangdong, China).

Figs 3A , 4A , 5A , Table 3

##### Holotype.

[FJIQBC 19280] Shell height 29.0 mm, shell width 52.5 mm, height of aperture 21.3 mm, width of aperture 27.0 mm, 24 April 2014, collected from the type locality.

##### Paratype.

[FJIQBC 19281–19293] 13 specimens: 2 empty adult shells, 11 live snails including 9 adults and 2 juveniles. Results of adult measurements: shell height 24.0–34.0 (27.40 ± 2.96) mm, width 49.8–59.5 (53.00 ± 2.77) mm, height of aperture 18.0–25.0 (20.75 ± 1.74) mm, width of aperture 22.0–31.0 (26.35 ± 2.57) mm, 24 April 2014, collected from type locality.

##### Type locality.

Kasuo, Lingyun, Guangxi, China (24°17'47.33"N, 106°39'6.53"E).

##### Etymology.

The name of the new species refers type locality.

##### Description.

*Shell*. Shell sinistral, large, slightly thin, semi-translucent, hard and fragile, flat globose. 4.75 whorls, the upper whorls increasing fast and slightly convex. Spire relatively low. Body whorl rapidly expanded, convex, with a weakly obtuse angulated margin at periphery. Shell fawn with countless light chestnut spiral bands. Spiral bands slender and dense below the periphery of body whorl, forming wide area of bands. Growth lines dense and thick on the surface. Apex quite blunt. Growth lines on protoconch visible when using 15× magnification. Suture line shallow. Aperture lunate, slightly descending in front view. Peristome reflected, sharp and white. Columellar lip reflected, slightly covering the umbilicus. Inner lip attached to the body whorl, forming translucent, thin and smooth callus. Umbilicus open and round. The first whorl can be seen through the umbilicus. Hump beside umbilicus absent.

*Soft body*. Foot hazel. Tentacles darker. White band from the head to the neck.

*Reproductive system*. Penis slightly swollen, short. Epiphallus long and thick. Penis retractor muscle very slender and long. Flagellum medium length, thick basally, tapering distally. Vas deferens long and thin. Vagina thick and slightly short. Bursa copulatrix oval. Pedunculus of bursa copulatrix quite long, expanded at basal half, while smooth and slender at the end. Inner penial wall supporting transverse, smooth, and dense pilasters proximally and several longitudinal, thin, curly, and widely-spaced pilasters distally. Verge conical and smooth, with eight transverse wrinkles basally. An obvious longitudinal crack on the verge, and six smooth and longitudinal pilasters with wide space in the crack. Verge opens laterally.

##### Ecology.

This species was found on limestone in Lingyun county of Guangxi province. It generally inhabits mountaintops with clouds and mists, but cannot be found at the foot of the mountain.

##### Remarks.

The key characters of *Camaena lingyunensis* sp. n. and the other eight sinistral *Camaena* species are presented in an identification key. This species is clearly different from other species, with a more oblate shape, lower spire, thinner and more fragile shell, and lighter colouration. The umbilicus of the new species is fully open, and the first whorl can be seen from the umbilicus, which significantly is distinguished from *Camaena poyuensis* (Zhou, Wang & Ding, 2016) and *Camaena obtecta* (Fischer, 1898) without an umbilicus, *Camaena inflata* and *Camaena connectens* (Dautzenberg & Fischer, 1906) with a narrow umbilicus, and *Camaena cicatricosa* and *Camaena detianensis* sp. n. with a semi-open umbilicus. The new species is similar with another sinistral snail, *Camaena seraphinica*, which also has a fully open umbilicus, but with a relatively higher and arched spire, wide and beautiful red spiral bands, and colour spots and no forged trace on the surface. Furthermore, the base region colour of the body whorl around the umbilicus is white. Differently, the new species has a flat and low spire with an inconspicuous forged trace, and contains countless slender spiral bands. The base region colour of the body whorl is the same as the shell surface with slender spiral bands.

Some sinistral *Camaena* species have the same features as the new species on penial wall, such as *Camaena hahni*, *Camaena poyuensis* and *Camaena inflata* (Ding et al. 2016), while the verge of these species is significantly different from the new species. The surface of verge of *Camaena hahni*, *Camaena poyuensis* and *Camaena inflata* all contains transverse or longitudinal microgrooves, but in the new species it is conical and smooth, and does not have any microgrooves except for a longitudinal crack, from which six longitudinal and smooth pilasters can be seen. There are six longitudinal and deep cracks on verge of *Camaena poyuensis*, but the cracks cannot be riven.

COI gene *p*-distances between this new species and the other seven sinistral species are 0.098–0.178 (Table 3). On phylogenetic tree, *Camaena lingyunensis* sp. n. is adjacent to *Camaena cicatricosa* and *Camaena detianensis* sp. n. However, the shells of the three species were greatly distinct as follows: (1) the fawn shell color of *Camaena lingyunensis* sp. n. is much lighter than the dark tawny shell color of *Camaena cicatricosa* and brown shell color *Camaena detianensis* sp. n. (2) The spire of *Camaena lingyunensis* sp. n. is low and flat, while the spires of the latter two are relatively high and arched (3) the *Camaena lingyunensis* sp. n. has countless light chestnut and slender spiral bands on the body whorl while *Camaena detianensis* sp. n. has no spiral bands (4) the umbilicus of *Camaena lingyunensis* sp. n. is fully open, while that of the latter two are semi-open.

#### Camaena detianensis

Taxon classificationAnimaliaStylommatophoraCamaenidae

Zhou & Lin sp. n.

http://zoobank.org/5F5B2C7E-954C-4644-8644-9873877959C5

Figs 3B , 4B , 5B , Table 3

##### Holotype.

[FJIQBC 18472] Shell height 38.5 mm, shell width 61.4 mm, height of aperture 22.5 mm, width of aperture 26.2 mm, 21 May 2013, collected from the type locality.

##### Paratype.

[FJIQBC 18473–18482] 10 specimens: all live snails including 4 adults and 6 juveniles. Shell height 34.2–40.4 (38.46±2.49) mm, width 55.0–62.5 (59.60±2.41) mm, height of aperture 19.6–23.9 (22.44±1.67) mm, width of aperture 24.2–28.1 (25.86±1.58) mm, 21 May 2013, collected from holotype locality.

##### Type locality.

Detian Falls, Daxin, Guangxi, China (22°51'29.54"N, 106°43'13.51"E).

##### Etymology.

Named for the type locality, adjective.

##### Description.

*Shell*. Shell sinistral, large, thick, solid, non-transparent and depressed-globular. 5.5 whorls, the front whorls increasing and convex rapidly. Spire arched. Body whorl expanding with an acute carina at periphery and a shallow groove-like depression above and below the carina, obviously near the aperture. Apex quite blunt. Growth lines on protoconch visible when using 15× magnification. Suture line deep. Surface dark brown and slightly red with obvious malleation. Spiral bands absent. The upper part of the periphery of body whorl with countless thick growth lines, convex, and the lower part smooth. Spire ribs below the periphery relatively obvious. Aperture lunate. Peristome reflected, white and not sharp. Columellar margin reflected. Umbilicus partly covered by reflected columellar lip. Inner lip attached to the body whorl tightly, forming translucent, smooth and thick callus. Umbilicus obvious and round. A hump beside umbilicus absent.

*Soft body*. Foot and tentacles are dark brown, and the head color is relatively light.

*Reproductive system*. Penis slightly long, slender and smooth. Epiphallus long and thin. Penis retractor muscle short, wide and flat. Flagellum very slender and long. Vagina smooth and slender. Vas deferens slender. Bursa copulatrix oval. Pedunculus of bursa copulatrix quite long, expanded at the base. Inner penial wall supporting several longitudinal, dense, and curly pilasters with narrow space. Verge long conical with many shallow, longitudinal and narrow pilasters. A shallow transverse microgrooves at the apical 1/3, surrounding the verge. A harelip-like crack at the end of verge. Verge opens terminally.

##### Ecology.

The species was found on limestone in Daxin county of Guangxi province. It generally lives in primeval forest or forest with a good ecological environment, and cannot be found in gardens near the forest. The population density of this new species in field is relatively low.

##### Remarks.

The key characters of *Camaena detianensis* sp. n. and the other eight sinistral *Camaena* are presented in the identification key. The carina and groove above and below the carina of *Camaena detianensis* sp. n. are typical features, which are different from other species of *Camaena* except for *Camaena hahni*. There is no obvious groove-like depression above and below the periphery of *Camaena hahni*.

The differences of shell between the new species and *Camaena cicatricosa* were as follows: (1) *Camaena detianensis* sp. n. is quite large, and the largest width can reach 62.5 mm, while the maximum width of shell of *Camaena cicatricosa* is less than 50 mm (2) The shell of this species is dark brown without spiral bands, while the shell of *Camaena cicatricosa* is yellowish brown, and contains many annular spiral bands (3) There is an acute carina at periphery of body whorl of the new species, and groove-like depression above and below the carina, but *Camaena cicatricosa* has no obvious carina.

The new species has similar appearance with *Camaena hahni*, both of which have semi-open umbilicus and a distinct carina at periphery of body whorl, but there still exist differences (1) *Camaena hahni* is relatively small, and the width of shell of mature specimen is generally between 45.0–47.0 mm. While the new species is very large, and the width of shell is generally between 55.0–62.5 mm (2) Compared to the new species, *Camaena hahni* has a sharper carina, but there is no obvious groove-like depression above and below the carina (3) The growth lines and spire ribs of *Camaena hahni* are thicker, and the shell surface is very rough, while that of the new species is relatively finer (4) The new species has thicker callus, while *Camaena hahni* has thinner callus.

A dissection of reproductive system shows that the pilasters of penis wall and verge shape of *Camaena detianensis* sp. n. is similar to that of *Camaena cicatricosa*, both of which have longitudinal and curly pilasters on the penis wall with narrow spaces, and the verge is conical with many longitudinal pilasters, while *Camaena hahni* has longitudinal and transverse pilasters on the penis wall, and verge is semicircle and small. There is an annular microgroove on verge, and a harelip-like crack at the end of verge in the new species, which is a diagnostic feature differing from other sinistral *Camaena* species.

COI gene *p*-distances between this new species and other seven sinistral species were 0.073–0.189 (Table 3). On the phylogenetic tree, this new species and *Camaena cicatricosa* are mutually sister groups, and *p*-distance of the two species is 0.073–0.086. According to above information, it is reasonable that the species is recognized as a new species (Criscione and Köhler 2014).

### Key to the sinistral *Camaena* species

**Table:** 

1	Umbilicus open	**2**
–	Umbilicus closed	**4**
2	Completely open umbilicus	**5**
–	Not completely open umbilicus	**3**
3	Semi-open umbilicus and no hump beside the umbilicus	**6**
–	Narrow umbilicus and a hump beside the umbilicus	**8**
4	No hump beside the umbilicus; many transverse microgrooves and few longitudinal deep groove on surface of verge	***Camaena poyuensis* (Zhou, Wang & Ding, 2016)**
–	A hump beside the umbilicus; many irregular curly grooves on surface of verge	***Camaena obtecta* (Fischer, 1898)**
5	No malleation on shell; a wide red band at periphery of body whorl	***Camaena seraphinica* (Heude,1890)**
–	Malleation on shell and countless chestnut slender spiral bands, forming a wide area of bands below periphery of body whorl	***Camaena lingyunensis* sp. n.**
6	Acute carina at periphery of body whorl	**7**
–	No obvious carina at periphery of body whorl	***Camaena cicatricosa* (Müller, 1774)**
7	Shell medium size; no obvious groove-like depression above and below the carina at periphery of body whorl; verge short and semicircle without a harelip-like crack	***Camaena hahni* (Mabille, 1887)**
–	Shell very large; shallow groove-like depression above and below the carina at periphery of body whorl; verge long and conical with a harelip-like crack	***Camaena detianensis* sp. n.**
8	Shell globose and thick; verge with transverse deep wrinkles basally and dense longitudinal microgrooves apically	***Camaena inflata* (Möllendorff, 1885)**
–	Shell depressed-globose; verge with longitudinal deep wrinkles only	***Camaena connectens* (Dautzenberg & Fischer, 1906)**

## Discussion

In the present study, two new species of sinistral *Camaena* were identified based on shell structure and colouration, reproductive system morphology, and molecular characteristics. *Camaena lingyunensis* sp. n. can be distinguished from other sinistral camaenids by the flat, thin, fragile, semi-translucent, and light coloured shell, especially the unique flat globose shape. The large shell, thick callus, acute carina at periphery of the body whorl and groove-like depression above and below the carina are key features of *Camaena detianensis* sp. n.

Genetic distance has been generally used for classification and determination of Camaenidae, such as the Australian camaenid *Kimberleytrachia* (0.055–0.161, Criscione and Köhler 2014), the Japanese camaenid *Luchuhadra* (0.003–0.205, Kameda et al. 2007), and the Taiwanese camaenid *Satsuma* (0.006–0.150, Wu et al. 2008). The *p*-distance between *Camaena lingyunensis* sp. n. and the other seven sinistral *Camaena* was significant, 0.098–0.178, as well as between *Camaena detianensis* sp. n. and the other seven sinistral *Camaena*, 0.073–0.189. All attain interspecific differentiation, and molecular phylogenetic analyses also support these two new species.

*Camaena detianensis* sp. n. and *Camaena cicatricosa* are closer in phylogeny and reproductive system dissection besides the shell morphology. While *Camaena detianensis* sp. n. and *Camaena hahni* have the similar shell. *Camaena hahni broti* (Dautzenberg & d’Hamonville, 1887) once was regarded as a subspecies of *Camaena hahni*, which is distributed in Nuy-Dong-Nay, Lang-Son, Vietnam. Due to the lack of specimens of *Camaena hahni broti* we did not compare it with the new species. However, from the pictures of syntypes it can be seen that *Camaena hahni broti* (MNHN-IM-2000-1848) and *Camaena hahni* (MNHN-IM-2000-1906) have the same size, morphology, and geographical distribution. Actually, Pilsbry considered *Camaena hahni broti* as a synonym of *Camaena hahni* in 1891. In the article of Ding et al. (2016), *Camaena cicatricosa ducalis* (Ancey, 1885), a subspecies of *Camaena cicatricosa*, was not revised due to lack of specimens. In this research, molecular comparison was not conducted either. According to literature records, *Camaena cicatricosa ducalis* was named based on a single specimen collected from Kouy-Yang-Fou (nowadays Guiyang), Guizhou. No further specimens were confirmed or recorded since its publication. The shell of *Camaena cicatricosa ducalis* (Ancey, 1885) with narrow umbilicus is quite large, shell width is 74 mm, but the maximum sinistral snail *Camaena detianensis* sp. n. is 62.5 mm. Some scholars have made great efforts to collect *Camaena cicatricosa ducalis* (Ancey, 1885), but failed (Ding et al. 2016). It is possible that this species has died out. On the other hand, *Camaena seraphinica* demonstrates great differences from the other sinistral *Camaena* by possessing a non-malleated surface, and white shell background with few wide bands; these characters are closer to Bradybaenidae in shell.

During the gradual lifting from the north Vietnam to the mid-west of Guangxi and then the Yunnan-Guizhou Plateau, the biological and geographical climate conditions changed complicatedly. The limestone landform is widely distributed in these areas and the complex environment has provided helpful conditions for life and reproduction of land snails. These areas have become the hot spots in research on biodiversity of land snails, and many new species have been found in recent years (Páll-Gergely et al. 2016; Páll-Gergely et al. 2015; Schileyko 2011; Nordsieck 2007). In our opinion, as research progresses, more and more new species of *Camaena* will be found in this area. Hence, a phylogenetic research based on morphology and molecular biology of *Camaena* is essential and urgent.

## Supplementary Material

XML Treatment for Camaena

XML Treatment for Camaena lingyunensis

XML Treatment for Camaena detianensis
